# Soliton bursts and deterministic dissipative Kerr soliton generation in auxiliary-assisted microcavities

**DOI:** 10.1038/s41377-019-0161-y

**Published:** 2019-05-29

**Authors:** Heng Zhou, Yong Geng, Wenwen Cui, Shu-Wei Huang, Qiang Zhou, Kun Qiu, Chee Wei Wong

**Affiliations:** 1 Key Lab of Optical Fiber Sensing and Communication Networks, University of Electronic Science and Technology of China, 611731 Chengdu, China; 2 Fang Lu Mesoscopic Optics and Quantum Electronics Laboratory, University of California, Los Angeles, CA 90095 USA; 3 Department of Electrical, Computer, and Energy Engineering, University of Colorado, Boulder, CO 80309 USA; 4 Institute of Fundamental and Frontier Sciences, University of Electronic Science and Technology of China, 611731 Chengdu, China

**Keywords:** Physics, Optics and photonics

## Abstract

Dissipative Kerr solitons in resonant frequency combs offer a promising route for ultrafast mode-locking, precision spectroscopy and time-frequency standards. The dynamics for the dissipative soliton generation, however, are intrinsically intertwined with thermal nonlinearities, limiting the soliton generation parameter map and statistical success probabilities of the solitary state. Here, via use of an auxiliary laser heating approach to suppress thermal dragging dynamics in dissipative soliton comb formation, we demonstrate stable Kerr soliton singlet formation and soliton bursts. First, we access a new soliton existence range with an inverse-sloped Kerr soliton evolution—diminishing soliton energy with increasing pump detuning. Second, we achieve deterministic transitions from Turing-like comb patterns directly into the dissipative Kerr soliton singlet pulse bypassing the chaotic states. This is achieved by avoiding subcomb overlaps at lower pump power, with near-identical singlet soliton comb generation over twenty instances. Third, with the red-detuned pump entrance route enabled, we uncover unique spontaneous soliton bursts in the direct formation of low-noise optical frequency combs from continuum background noise. The burst dynamics are due to the rapid entry and mutual attraction of the pump laser into the cavity mode, aided by the auxiliary laser and matching well with our numerical simulations. Enabled by the auxiliary-assisted frequency comb dynamics, we demonstrate an application of automatic soliton comb recovery and long-term stabilization against strong external perturbations. Our findings hold potential to expand the parameter space for ultrafast nonlinear dynamics and precision optical frequency comb stabilization.

## Nonlinear optics: Keeping solitons warm and stable

Ultrafast optical states called solitons can be prevented from thermally breaking down by carefully heating them with a laser, researchers in the US and China show. Solitons are optical fields that exist in isolation, like smoke rings in air or bubbles in water, and they could greatly improve precision laser measurements and spectroscopy. However, it is difficult to maintain robust soliton states due to nonlinear thermal effects that cause them to break down. Heng Zhou at UESTC, Chee Wei Wong at UCLA, and co-workers generated solitons by directing a ‘frequency comb’ source (comprising discrete, equally-spaced laser lines) onto a silicon nitride optical microcavity. Crucially, they employed a second laser to provide heating to the system and suppress the thermal nonlinearities. This enabled smooth transitions between useful soliton states, while avoiding chaotic intermediate states.

## Introduction

Dissipative Kerr solitons (DKS) can be obtained in optical cavities through the double balancing of cavity loss with third-order nonlinear parametric amplification and cavity dispersion with self-phase modulation (SPM)^1–3^. In particular, DKS generated in high finesse microresonators associated with a Kerr frequency comb has attracted considerable interest, as it simultaneously gives rise to ultrashort mode-locked pulses^4–6^ and broadband comb spectra^7–10^ with good intrinsic stabilization potential^11^ and a smooth envelope^12^. Moreover, DKS microcombs not only exhibit abundant physical dynamics^13–26^ but also demonstrate immense practical prospects ranging from high capacity fiber transmission^27,28^, an ultra-stable microwave source^29^, and a photonic frequency synthesizer^30^, to precision laser metrology and spectroscopy^31–35^.

The robust generation of DKS, especially the single soliton state, however, remains difficult due to the sizable cavity thermal nonlinearity^1,36^, which prevents the pump laser from entering the parameter space where the dual-balanced solitary waveforms exist. The current strategy used for DKS formation is based on the fast scanning of pump laser frequency to overtake the slower dragged thermal response in a rapidly changing intracavity waveform and stopping the pump as close to the optimal condition as possible^6,15,37–41^. This scheme sets the microresonator to a delicate thermal equilibrium with complex detuning conditions of the intracavity light fields;^1,15^ consequently, one requires rigidly conducted pump frequency-sweeping^39–41^ and “power kicking”^5,38^, during which precision control of the soliton state is cumbersome^38,40^. Moreover, such a fast pump scanning approach contains an intrinsic prerequisite that, to keep the cavity in thermal equilibrium, the intracavity light fields must sustain effective blue-detuning even after the pump laser enters the red-detuning resonance region^1,40^. This prerequisite results in high pump power operation and, thereby, a chaotic stage prior to accessing the low-noise soliton states^42–44^. The resulting DKS pulse number and its comb envelope thus vary from measurement to measurement. Consequently, thermal compensation approaches have been investigated, such as cavity optomechanics with dual lasers^45^ and stabilization of the cavity mode spacing^46^.

Here, we demonstrate, via an auxiliary laser heating approach, an expanded operating phase space for the nonlinear transitions of the Kerr frequency comb along with unique dynamics. Through separation of the cavity thermal nonlinearities from the Kerr dynamics, we first uncover an existence range in the inverse-sloped Kerr soliton evolution featuring a diminishing soliton energy with increasing pump detuning, arising from a unique balance between soliton peak power and pulse width. Second, we report deterministic transitions from Turing-like comb patterns directly into a dissipative Kerr soliton singlet pulse, avoiding the subcomb overlaps and thereby bypassing the chaotic states in the formation dynamics. Third, we illustrate dissipative Kerr soliton bursts arising from background noise via the red-detuning pump entrance. Aided by these new findings, we implement an automatic soliton recovery algorithm against strong external perturbations.

## Results

### Stable DKS generation via auxiliary laser heating

**Fig. 1:**
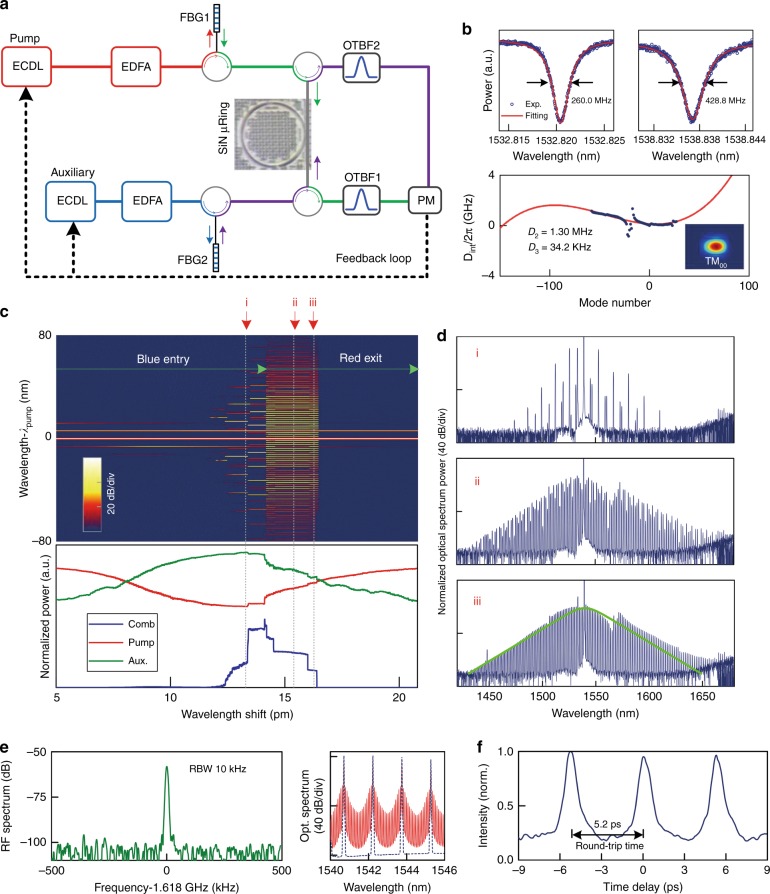
Auxiliary-assisted frequency comb and stable DKS generation with pump-cavity blue-sided entrance. **a** Schematic for the experimental setup. Two ECDLs (one used as the auxiliary laser and the other as the pump laser) are amplified and launched into a Si_3_N_4_ microring resonator from opposite directions. The auxiliary laser is preset in the blue-detuning regime of one cavity mode, which maintains the cavity thermal stability and escorts the pump to stably traverse an entire cavity linewidth. EDFA: erbium doped fiber amplifier; FBG: fiber Bragg grating; PM: power meter; OTBF: optical tunable bandpass filter. **b** Upper panel: measured linewidth for two cavity resonances centered around 1532.8 nm and 1538.8 nm, from where the auxiliary and pump lasers enter the cavity. The two resonances have a loaded *Qs* of ≈750,000 and 450,000, respectively. Lower panel: measured (blue dots) and FDTD simulated (red solid line) dispersion *D*_int_ = *ω*_*μ*_ ­ *ω*_*0*_ *­ μD*_1_ of the fundamental TM_00_ mode of the microcavity. Here, *ω*_0_ is the angular frequency of the pumped cavity mode, *ω*_*μ*_ is the angular frequency of the *μ*-th cavity mode relative to *ω*_0_, and *D*_1_ is the FSR measured at *ω*_0_. The inset shows the simulated mode profile for mode TM_00_. **c** Measured frequency comb spectrum (upper) and power evolutions (lower) as a 1.0 W c.w. pump laser adiabatically scans from the blue- to red-detuning regimes of a cavity mode. The pump scan is conducted with increasing step wavelength, with a 40 fm (5 MHz) step size and a delay of 0.1 s after each step. The auxiliary laser is operated at similar intensities. The pump and auxiliary laser dynamics counter-balance thermal influences on the microcavity, resulting in a pump power evolution with little thermal hysteresis. **d** Optical spectral snapshots of the generated Kerr frequency comb with the (i) low-noise subcombs state, (ii) multiple DKS state, and (iii) singlet DKS state. The RF spectra are consistently at the noise floor for all three comb states. **e** Beat note measurement of the singlet DKS frequency comb (left panel), using cross-phase-modulation sidebands to reduce the initial comb mode spacing (right panel). **f** SHG-based autocorrelation measurement for the singlet DKS frequency comb

Figure 1a illustrates our experimental setup. A Si_3_N_4_ microring cavity with a width-height cross-section of 2000 × 800 nm^2^ and a loaded *Q*-factor of ≈500,000 is utilized for frequency comb generation. More than ninety resonant frequencies of the TM_00_ mode are recorded via a Mach–Zehnder interferometer (MZI)-based optical sampling technique^47^ from which the cavity group velocity dispersion (GVD) is retrieved. The measured dispersion matches well with finite-difference time-domain (FDTD) numerical simulations, as shown in Fig. 1b, with a second-order GVD (*D*_*2*_) of 1.30 MHz and third-order dispersion (*D*_*3*_) of 34.2 kHz obtained. GVD discontinuity of the TM_00_ mode caused by mode-crossing with another spatial mode family is observed^4,12^, the influence of which will be discussed in the below sections.

Our approach uses two external cavity diode lasers (ECDL), which are amplified and launched into the microcavity from opposite directions (Fig. 1a). First, an auxiliary laser (*E*_aux_) is frequency tuned into a resonance (≈1532.8 nm in our experiment) following the traditional self-thermal locking trajectory and is stopped near the resonance peak but still kept blue-detuned. Subsequently, a pump laser (*E*_pump_) is tuned into another resonance (≈1538.8 nm) in the counter-propagating direction. Due to the effect of *E*_aux_, the evolution of *E*_pump_ is significantly modified from the conventional pathway. In particular, as *E*_pump_ is tuned into resonance from the blue side, all the resonances are heated and thermally pushed to longer wavelengths, which displaces *E*_aux_ from its resident resonance and cools the microcavity in counter-balance. Likewise, when *E*_pump_ leaves the cavity resonance from the red side and cools the cavity, *E*_aux_ will re-enter its resident resonance and in turn heat the cavity (see Supplementary Information Section I). By aptly configuring the power and frequency for *E*_aux_, the heat flow caused by *E*_pump_ can be largely balanced out, keeping the cavity temperature and all cavity resonances approximately unchanged. This allows the pump laser to be stably tuned across the entire resonance with minimized thermal behavior. Furthermore, since *E*_aux_ and *E*_pump_ are counter-propagating light waves with different detunings, the Kerr-nonlinearity cross-phase modulation (XPM) between them induces a slowly varying nonlinear detuning offset^48^, which retards the change in the effective pump detuning (similar to thermal counter-dragging) and in turn increases the detuning range wherein DKS can be accessed (see Supplementary Information Section VIII).

As shown in Fig. 1c, by implementing a dual-driven scheme, the experimentally measured power transmissions, *E*_pump_ and *E*_aux_, clearly illustrate their counter-balanced contributions to the cavity thermal behavior. We observe that when the pump traverses from the blue-detuning (*λ*_i_) to the red-detuning (*λ*_iii_) regime, stable comb spectra can be formed, as shown in Fig. 1d-(i)–d-(iii). Specifically, when the *E*_pump_ is blue-detuned (*λ*_i_), subcombs with well separated line doublets (i.e., spacing multiple FSR) are generated^42^, as shown in Fig. 1d. When the pump enters the red-detuned regime (*λ*_ii_), the oscillator achieves gap-free single FSR comb spectra resembling multiple DKS waveforms. Upon further tuning the pump toward the red-sided regime, we observe step-like discrete soliton annihilations, and eventually, a single soliton microcomb with a *sech*^*2*^ envelope is achieved (*λ*_iii_). The RF spectra are consistently at the noise floor for all three comb states. Moreover, the beat note of the single DKS comb is measured via a cross-phase modulation method (detailed in Supplementary Information Section II). As shown in Fig. 1e, the beat note is much narrower than the pump laser linewidth (≈500 kHz), confirming the good coherence of the DKS microcomb. We also conducted second-harmonic generation (SHG)-based autocorrelation measurement of the single DKS comb. As displayed in Fig. 1f, clear pulses are obtained with a temporal width of ≈650 fs and period of 5.2 ps, consistent with the cavity round-trip time and confirming singlet DKS operation (detailed in Supplementary Information Section IV). Our auxiliary-driven approach relaxes the prior fast sweeping rate requirement for the pump laser for DKS formation, allowing ease in setting the various DKS states at different detunings for dynamical exploration.

### Existence of a DKS regime supporting diminishing soliton pulses

A noteworthy feature of the auxiliary-assisted DKS evolution is observed in Fig. 1c. Specifically the step-like comb power evolution (blue line) exhibits an inverse negative slope as a function of pump detuning. In other words, as the pump detuning increases in this experiment, the overall DKS energy decreases, revealing a diminishing soliton regime. Such regimes were absent in prior DKS generation schemes realized via fast-pump-tuning, which always has an increasing intracavity energy with larger pump detuning (i.e., positive-slope soliton stairs) to maintain soliton self-thermal-locking^1,12,15,38,39^. Along with the diminishing soliton energy with pump detuning, in our studies, we also observed appreciable relaxation of the pump power requirement for DKS formation. As shown in Fig. 3a, a single DKS comb state was generated with 0.15 W of on-chip pump power, which is much smaller than prior DKS generation with a similar quality factor^6,24^.

**Fig. 2:**
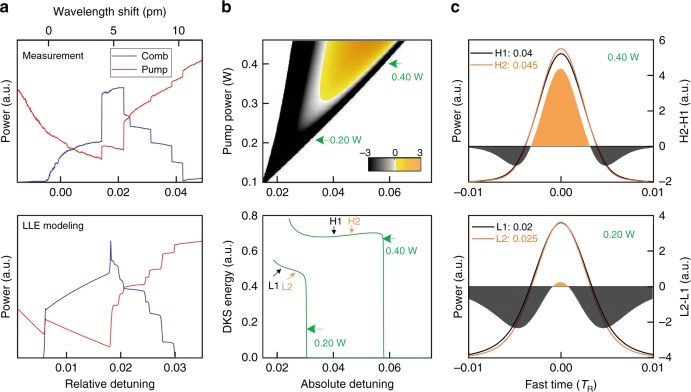
Existence of a DKS regime with effectively red-detuned soliton dynamics. **a** Comparison of experimental and theoretical pump power transmission and Kerr comb power evolution as a function of pump detuning. In this measurement, the launched pump power is 1.25 W. Good agreements are achieved between measurement and theory, especially for the inverse negative-slope comb power stairs. **b** Numerically mapped DKS existence range with identified detuning of the Kerr comb, as determined by the ratio Δ*P*_comb_/Δ*δ*. The lower panel gives two examples of an increasing (0.40 W) and diminishing (0.20 W) soliton regime. L1 and L2 (H1 and H2) denote two soliton pulses extracted from the DKS energy curve generated for a pump power of 0.2 W (0.4 W); the corresponding detunings are listed in **c**. **c** Close-up soliton waveform evolution versus pump detuning in the increasing (upper) and diminishing (lower) soliton regimes

To discern these new phenomena, we conduct a Lugiato-Lefever equation (LLE) simulation using our experimental parameters (detailed in the Materials and Methods). Figure 2a compares the experimental and theoretical pump and comb power evolutions, which agree well with each other; both exhibit inverse-slope comb power steps. To verify DKS existence in this new range, in Fig. 2b, we illustrate the calculated comb power versus pump detuning (Δ*P*_comb_/Δ*δ*) in a wide parameter space using the pulse seeding method^14,26^. At a relatively high pump power, DKS evolution has a typical positive slope as shown in the yellow to orange region. In contrast, at lower pump powers, a new diminishing soliton regime with an inverse-slope is clearly identified by the gray to black regions in this map.

The underlying physical mechanism for different DKS regimes lies in the subtle variation of the DKS pulse evolutions. As shown in Fig. 2c, with the conventional higher pump power, the increase in soliton peak power is more prominent than the decrease in soliton pulse width, so that the overall pulse energy increases with pump detuning. In contrast, when the pump power is lowered with our auxiliary laser heating approach, the order of importance between the soliton peak power and pulse width is reversed, and the overall soliton energy decreases with increasing detuning (complementary measurements and analytical formula are detailed in Supplementary Information Sections V and VI). It is notable that this new diminishing soliton regime with a relaxed extrinsic pump power requirement can help to expand the DKS existence range in relatively low-finesse microcavities^7^, supporting octave-spanning Kerr combs with ultrashort DKS pulse widths and low energies^40^, enabling low repetition rate DKS in long microcavities^49^, and allowing deterministic DKS formation, as presented below.

### Deterministic DKS formation without chaotic stages

**Fig. 3:**
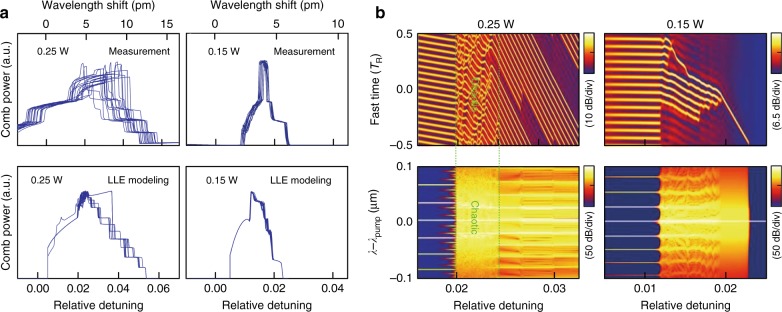
Deterministic singlet DKS formation without the intermediate chaotic stage. **a** Experimental (upper) and numerical (lower) investigations of DKS repeatability with different pump power levels. The experimental powers are estimated considering an ~7 dB pump insertion loss from the EDFA to the ring cavity. The LLE simulation is seeded by random noise and includes the measured GVD discontinuity from local mode interactions. The right panel shows that when a relatively low pump power is used, deterministic DKS formation dynamics are recorded both in experiment and in simulation. **b** Modeling of the intracavity waveforms (upper) and comb spectra (lower) at different pump power levels. It is clearly seen that a chaotic intermediate stage exists for 0.25 W of pump power, which is absent for 0.15 W of pump power, allowing a direct transition from a Turing pattern to a single-DKS state

In addition to an expanded existence regime, the auxiliary-assisted microcavity allows the robust evolution of the frequency comb into the singlet DKS state. Figure 3a shows twenty example repeated measurements of the time-trace for the comb power evolution, each of which evolves to a singlet DKS. This phenomenon is distinct from prior demonstrations using either fast-swept pump or power-kicking schemes^5,15,38^ in which the DKS state has a statistical success probability, and the eventual DKS pulse numbers are not deterministically controlled. As reported in recent studies^50,51^, this phenomenon relies on the mechanism that the mode interaction-induced GVD discontinuity can suppress or enhance individual comb lines^18,50^, giving rise to an oscillatory background attached to each DKS, akin to the effect of Kelly sidebands in mode-locked fiber lasers^52^. Such oscillating pump backgrounds facilitate mutual soliton interferences^14^, and finally only one last DKS survives as the pump detuning approaches the maximum value (i.e., the pump power approaches a minimum value) before the comb collapses^50^. To verify this phenomenon, we conduct LLE modeling using the measured GVD data that include discontinuities from local mode interactions (blue dots in Fig. 1b)^53^. As shown in Fig. 3a (bottom left panel), twenty independent LLE simulations initiated by random noise seeds all result in a singlet DKS state, agreeing well with our measurements.

Meanwhile, in Fig. 3a, we observed that for each measurement, the comb power evolution paths to the singlet DKS state still undergo random changes. This randomness arises from the overlaps of the subcomb family during Kerr comb generation, which includes a chaotic stage in the intracavity waveform formation and sets a statistically random initial condition for the subsequent DKS convergence^1^, as illustrated in Fig. 3b. Usually in the fast-pump-tuning scheme, strong pump intensity is required to maintain self-thermal locking of the DKS state, and the dynamical route has to go through this chaotic regime^44,54^. Our auxiliary laser heating approach reduces the requirement on the pump intensity for DKS formation, as shown in Fig. 2b. This allows us to further reduce the pump intensity and explore the direct transition from a stable Turing pattern (i.e., primary comb lines) to the low-noise DKS state, avoiding the chaotic stage.

Figure 3a (top right panel) shows the measured comb power evolution to the single DKS state at 0.8 W of launched pump power (0.15 W of on-chip power, considering 7.0 dB attenuation during pump delivery). Strikingly, almost identical comb power trances are experimentally recorded in twenty independent DKS transition measurements (minor discrepancies are due to laser wavelength sweep repeatability fluctuations), all of which result in a singlet DKS without exception. Such dynamics are also captured in the LLE modeling, as shown in the lower right panel of Fig. 3a, confirming the deterministic formation of the single DKS comb without intermediate chaos. Figure 3b shows the numerically resolved spectral and temporal evolution of the DKS comb with a pump power of 0.15 W. We observe that, due to the relatively low pump power, the Kerr comb spectrum exhibits no subcomb overlap when the pump is blue-detuned. Subsequently the pump directly enters the red-detuning side without triggering subcomb spectral overlap. Thus, this intrinsically allows the temporal Turning pattern to directly and deterministically transit into the DKS state without a chaotic intermediate stage (the latter is typically formed from the subcomb dynamics). As the pump detuning further increases, multiple solitons step-wise annihilate and the singlet DKS state is reached aided by the GVD discontinuity^50,51^.

### Observation of DKS bursts with red-detuned pump entrance

**Fig. 4:**
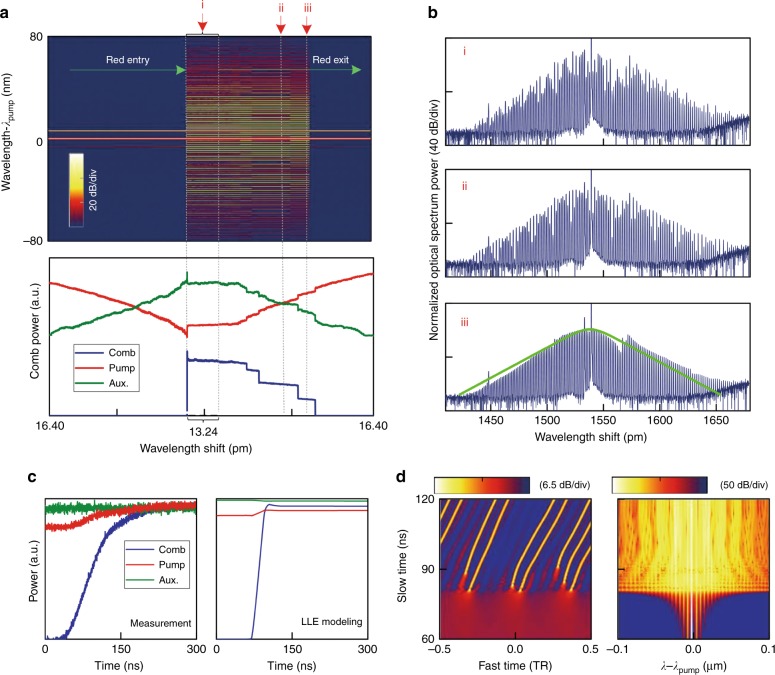
Spontaneous DKS burst with pump-cavity *red*-sided entrance. **a** The measured Kerr comb spectra (upper) and intracavity laser power evolutions (lower) generated using a 1.0 W pump laser stably scanning from red- to blue-detuning. The pump scan is conducted with a decreasing step wavelength, with a step size of 40 fm (≈5 MHz) and a delay of 0.1 s after each step. The pump laser scan is paused at 13.24 pm (relative shift) for a few seconds, and deliberately returned back to 16.40 pm to incur typical discrete soliton annihilations, which provides evidence for soliton formation during the DKS burst. **b** Optical spectral snapshots for a DKS burst (i) and subsequent discrete DKS breakdown (ii and iii). The RF spectra are consistently at the noise floor for all three comb states. **c** Measured (left) and LLE simulated (right) real-time intracavity power evolutions during the process of a DKS burst. Soliton pulses appear and stabilize within ≈50 ns, during which the auxiliary laser power (i.e., cavity resonance) hardly changes, indicating that intracavity energy is conserved. **d** Waveform and spectrum evolution from the LLE simulation in **c** illustrating a close view of spontaneous DKS burst from the perturbed pump background

For conventional Kerr comb generation, the pump laser always enters cavity resonance from the blue-detuning regime so as to acquire self-thermal locking^36^. As discussed above, the auxiliary laser heating scheme dispenses with canonical self-thermal locking and allows red-side pump entrance. This is demonstrated in Fig. 4a; we first configure a blue-detuned auxiliary laser and then let the pump laser enter the cavity mode from the red-detuning regime. Intriguingly, we observe sharp *DKS burst* dynamics directly from the continuum background noise (*λ*_i_) in which a multiple soliton comb state with low RF noise is generated (Fig. 4b). To validate the soliton nature of the burst intracavity waveform, we then tune the pump frequency out of resonance from the red side and, as expected, typical step-like soliton annihilations are detected (*λ*_ii_ and *λ*_iii_).

Figure 4c displays the fast DKS burst dynamics measured with a real-time oscilloscope; it is seen that the burst process is completed in approximately 50 ns, within which the cavity thermal response and pump frequency tuning are negligible, indicating the spontaneous nature of the DKS burst. LLE modeled burst dynamics illustrate remarkable agreement with experimental traces (Fig. 4c), allowing us to understand the detailed kinetics. In particular, when the pump laser enters the cavity from the red-detuning regime, SPM incurred by the increasing intracavity pump power redshifts all the cavity resonance, letting the cavity resonance and pump laser move towards each other. Resultantly, the pump laser builds up quickly and triggers Kerr comb generation after reaching the parametric oscillation threshold. Meanwhile, the formation of Kerr comb lines causes a decrease in the intracavity pump power and a concomitant SPM resonance redshift; conversely, the pump laser leaves the resonance from the red-detuning side, causing an iterative decrease in pump power. That is, the pump laser goes in and out of the cavity mode as a spontaneous chain reaction (since the pump frequency is fixed), leaving only an excited Kerr comb waveform, which spontaneously develops into DKS pulses as the pump eventually stabilizes in the red-detuning regime. Moreover, LLE modeled intracavity dynamics (Fig. 4d) give a clear visualization of the DKS burst mechanism: as the pump enters from the red-detuning side, even slight fluctuations within the pump background (e.g., caused by GVD discontinuity) can ignite the chain reaction pump movement, causing sudden soliton appearance from the seemingly flat pump background.

## Discussion

### DKS recovery against external perturbations

**Fig. 5:**
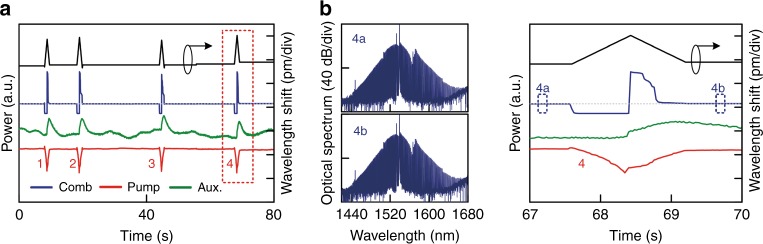
Demonstration of singlet DKS frequency comb recovery against external perturbations. **a** The left *y*-axis shows the laser power evolutions during DKS recovery against four externally enforced perturbation events. The right *y*-axis shows the wavelength shift of the auxiliary laser for regenerating the singlet soliton comb through spontaneous bursts and deterministic singlet formation. **b** Zoom-in dynamics for the example fourth perturbation and recovery event, and comb spectral snapshots before (left top) and after (left bottom) successful soliton recovery. The dashed gray lines in **a** and **b** indicate that the overall frequency comb power is precisely reset after each recovery event for the consistent low-noise singlet DKS comb state

Enabled by a red-pump-entry spontaneous DKS burst and deterministic generation, Fig. 5 demonstrates the effective singlet DKS recovery against strong external perturbations. As noted in prior studies, the DKS state can be vulnerable to pump laser instabilities including frequency noise, frequency drift and power fluctuations, requiring either comb power or pump detuning feedback^11,37,46,55^. To enable long-term operation for the singlet DKS, we thus measure the total comb power as an error signal to control the auxiliary laser wavelength for protected recovery of the DKS state against external variations. We implement a physical control algorithm as follows: when the comb power becomes smaller than a preset value corresponding to the singlet DKS state, we assume that the target state is lost; then, the auxiliary laser is tuned closer to the resonance peak while thermally red-shifting all resonances, and correspondingly, we let the pump re-enter from the red-detuned regime and trigger a DKS burst. Conversely, on the other side, when multiple solitons are regenerated with higher power than that associated with the singlet DKS, we decrease the auxiliary laser wavelength to blue-shift the cavity resonance and, correspondingly, let the pump leave the resonance until the singlet DKS comb power is restored.

Figure 5 illustrates the experimentally measured singlet DKS recovery dynamics, which are triggered by two mechanical hard vibrations on the pump and auxiliary lasers, respectively (labelled as events “1” and “2”), and two ambient acoustic disturbances (labelled as events “3” and “4”) – each of which are severe enough to drop the operating DKS microcomb. To illustrate the singlet DKS recovery, the wavelength shift of the auxiliary laser is simultaneously read out, as shown as the black line in Fig. 5. The pump, auxiliary and comb powers are shown as the red, green and blue lines, respectively. We observe that the singlet DKS comb state, characterized by the comb power, is successfully recovered from each of the four externally enforced perturbation events, with the close-up slow-time dynamics of the fourth event shown in Fig. 5b. When a strong acoustic shock is applied to both the pump and auxiliary lasers in event “4”, the comb power shows a fast decrease, indicating the collapse of all DKSs. At this stage, the cavity modes do not go back to their cold resonances due to the presence of the auxiliary laser that resides on the blue-detuned regime; thus, the resonances remain approximately unmoved. Subsequently, based on the negative feedback recovery algorithm, the decreased comb power leads to an increase in the wavelength of the auxiliary laser (black curve in Fig. 5), which thermally redshifts all cavity resonances and drives the pump laser onto resonance again from the red-detuned side, thus causing the spontaneous DKS burst to recover the comb. The auxiliary laser wavelength is then automatically decreased again to regain singlet DKS operation. We note that the auxiliary laser wavelength shift is all within 2 pm for successful DKS recovery, highlighting the efficacy of our recovery approach. We test our spontaneous burst and deterministic DKS approach against different perturbation strengths, and in each instance, the singlet DKS comb is recovered as long as the auxiliary laser keeps its thermal lock on the microcavity.

In conclusion, assisted by an auxiliary-laser, we demonstrated for the first time the separation of thermal nonlinearity from Kerr nonlinearity in microcavities. Counterbalancing the thermal nonlinearity, we uncover unique stable DKS formation routes in an expanded existence range featuring inverse-sloped soliton stairs. We also observed a direct transition from Turing-like patterns into a DKS state avoiding chaotic stages, enabled by the use of unprecedentedly low (normalized) pump power. Furthermore, via a red-detuned pump entrance, we demonstrated the existence of DKS bursts in which the soliton state is formed at the first stable instance directly through an instantaneous parametric modulation instability state. With the expanded existence range and precise deterministic control of the DKS states, we illustrate the robustness of our singlet DKS state with the application of dynamical feedback recovery against external perturbations in the frequency comb. This study enriches the physical understanding of dynamical Kerr solitons in dissipative nonlinear cavities and provides a new architecture for mesoscopic mode-locked frequency combs.

## Materials and methods

### Theoretical model for numerical simulations

The LLE model with a cavity thermal effect and auxiliary laser is expressed as follows:^14,56^

1 $$\begin{array}{l}T_R\frac{{\partial E_{\mathrm{comb}}(t,\tau )}}{{\partial t}} = \left[ { - \alpha - i\left( {\delta _{\mathrm{{pump}}} - \delta _{\mathrm{thermal}}} \right) + iL\mathop {\sum}\limits_{k \ge 2} \frac{{\beta _k}}{{k!}}\left( {i\frac{\partial }{{\partial \tau }}} \right)^k}\right.\\\left.+ i\gamma L\left( {\left| {E_{\mathrm{comb}}} \right|^2{\mathrm{ + 2}}P_{\mathrm{aux}}} \right) \right]E_{\mathrm{comb}}(t,\tau ) + \sqrt \theta E_{\mathrm{pump}}^{in}\end{array}$$

2 $$\begin{array}{l}T_R\frac{{\partial E_{\mathrm{aux}}(t,\tau )}}{{\partial t}} = \left[ { - \alpha - i\left( {\delta _{\mathrm{aux}} - \delta _{\mathrm{thermal}}} \right) + iL\mathop {\sum}\limits_{k \ge 2} \frac{{\beta _k}}{{k!}}\left( {i\frac{\partial }{{\partial \tau }}} \right)^k}\right.\\\left.+ i\gamma L\left( {\left| {E_{\mathrm{aux}}} \right|^2 + 2P_{\mathrm{comb}}} \right) \right]E_{\mathrm{aux}}(t,\tau ) + \sqrt \theta E_{\mathrm{aux}}^{in}\end{array}$$

3 $$\frac{{\partial \delta _{{\mathrm{thermal}}}}}{{\partial t}} = \frac{{\mathrm{1}}}{{C_p}}\left[ {\alpha _T\frac{{\mathrm{Q}}}{{Q_{abs}}}\left( {P_{\mathrm{comb}} + P_{\mathrm{aux}}} \right) - K\delta _{{\mathrm{thermal}}}} \right]$$

In Eq. (2), *T*_R_ is the round-trip time of the Si_3_N_4_ microcavity; *t* is the slow time describing the evolution of the intracavity comb field *E*_comb_ (*t*, *τ*) and auxiliary laser field *E*_aux_ (*t*, *τ*) at the scale of the cavity photon lifetime; *τ* is the fast time defined in a reference frame moving at the light group velocity in the cavity; *α* *=* 0.009 is the cavity decay per roundtrip; and *δ*_pump_ and *δ*_aux_ are the detunings of the pump and auxiliary laser, respectively; *γ* *=* 1.0 m^−1^W^−1^ is the nonlinear coefficient; *θ* *=* 0.009 is the coupling rate from the bus waveguide to the ring cavity; *β*_*k*_ is the *k*-th order dispersion, which utilizes the combinational data from both measurement (the −40th to 40th modes) and FDTD simulations (other modes), as shown in Fig. 1b. In the model, *α*, *θ*, and *β*_*k*_ are assumed to be identical for the pump and auxiliary lasers. As the comb and auxiliary laser fields counter propagate, XPM between them is subjected to their average power *P*_comb_ and *P*_aux_^48^, imposing a slowly varying nonlinear detuning offset to each other. It is notable that, as can be seen from Equations 1 and 2, when the pump laser enters red-detuning for DKS formation, the XPM induced by the auxiliary laser (2*iγLP*_aux_) counter balances the increase in the pump detuning *δ*_pump_ and decreases the self-phase modulation of the pump laser (contained in 2*iγL*|*E*_comb_|^2^), which in turn retards the increase in the effective pump detuning. That is, XPM by the auxiliary laser can expand the pump frequency range where DKS can be accessed, in a similar fashion to that in thermal dynamics (see Supplementary Information Section VIII for more details). Equation (3) above describes the cavity thermal dynamics, where *α*_*T*_ is the temperature coefficient of the detuning shift (1/^0^*C*); *C*_*P*_ is the thermal capacity (*J*/^0^*C*); *K* is the thermal conductivity (*J*∙*s*^−1^∙^0^*C*^−1^) of the microcavity to the atmosphere; *Q* and *Q*_abs_ are the loaded and intrinsic quality factors. respectively^36^. By fitting the experimentally measured pump transmissions, we have (*α*_*T*_*Q*)*/*(*Q*_*abs*_*C*_*P*_) = 5.0e-6 *J*^−1^ and *K/C*_*P*_ *=* 7×10^−4^ *s*^−1^. We note that the parameters *Q/Q*_*abs*_ and *K* are set to be larger than the conventional values in order to simulate the DKS formation process within a reasonable time^15^. To study the comb dynamics, Eqs. (1–3) is resolved via a split-step Fourier method with a step size of *T*_R_ (the detailed influences of the thermal effect and auxiliary laser are presented in Supplementary Information Section VII).

To acquire the existence range and effective detuning for DKS within the parameter space of pump power and detuning *δ*_pump_, we adopt a singlet soliton seeding method using the LLE model. This is achieved by starting the simulation with a hyperbolic secant pulse as the initial intracavity waveform, whose width and peak power are approximately configured according to the analytical solution^1,14^. First, we let the initial pulse numerically evolve under a fixed pump detuning for 10,000 roundtrip times (≈328× of the cavity photon lifetime) to find out the smallest detuning *δ*_0,m_ that allows the initial pulse to become a DKS without incurring parametric oscillation. This value for *δ*_0,m_ is the lower limit of the DKS existence range for the corresponding pump power level. Afterwards, a pump scanning simulation is conducted for the initial pulse, by using *δ*_0,m_ as the starting value and linearly increasing it at a rate of 3e-6/*T*_R_ until the DKS pulse vanishes. Δ*P*_comb_/Δ*δ* is recorded for each incident pump power, from which the DKS effective detuning condition is clearly revealed by the sign of the derivative curves, as shown in Fig. 2b.

## Supplementary information

supplementary file
