# Supplementary material for: Soliton bursts and deterministic dissipative Kerr soliton generation in auxiliary-assisted microcavities
Source: Light Sci Appl. 2019 May 29;8:50. doi: 10.1038/s41377-019-0161-y (PMC6538660; doi:10.1038/s41377-019-0161-y)
Supplement: Supplementary file 1 — supplementary file [file 41377_2019_161_MOESM1_ESM.docx]

**Supplementary Information for**

**Soliton** **bursts and deterministic dissipative Kerr soliton generation in auxiliary-assisted microcavities**

Heng Zhou^1, *, †^, Yong Geng^1, *^, Wenwen Cui^1^, Shu-Wei Huang^2, 3^, Qiang Zhou^4^, Kun Qiu^1^, and Chee Wei Wong^2^, ^‡^

^1^ Key Lab of Optical Fiber Sensing and Communication Networks, University of Electronic Science and Technology of China, Chengdu 611731, China

^2^ Fang Lu Mesoscopic Optics and Quantum Electronics Laboratory, University of California, Los Angeles, CA 90095, USA

^3^ Department of Electrical, Computer, and Energy Engineering, University of Colorado, Boulder, Colorado 80309, USA

^4^ Institute of Fundamental and Frontier Sciences, University of Electronic Science and Technology of China, Chengdu 611731, China

*equal contribution

Corresponding author: † zhouheng@uestc.edu.cn ; ‡ cheewei.wong@ucla.edu

**I. Configuration of auxiliary laser power and frequency**

Figure S1a shows the schematics of the auxiliary laser heating method, which clearly illustrates the mechanism how cavity heat flow caused by the auxiliary and pump laser keeps balanced, as described in the main text (a video demonstration can be find as supplementary information 2). Figure S1b shows the benchmark measurement of auxiliary laser assisted comb generation method. It is shown that, when pump power is set under parametric oscillation threshold, auxiliary laser cancels out the thermal pulling induced by the pump laser, and an ideal Lorentz resonance lineshape is obtained for both the blue- and red-side pump entries. Moreover, for stable DKS generation (pump power beyond parametric oscillation threshold), the power and wavelength of the auxiliary laser should be carefully configured to achieve a counterbalanced heat flow (with heating from both the pump and auxiliary lasers). When the intracavity power of auxiliary laser is too small (29.0 dBm and 30.0 dBm), we can see an abrupt decline of pump power when it crosses into the red-detuning regime (Figure S1c and S1d), indicating that the cavity heating caused by auxiliary laser cannot smoothly compensate the cavity cooling induced by the pump laser [S1]. We then gradually increase the intracavity auxiliary laser power by either increasing the launched power or decreasing the detuning value, until a smooth pump transmission curve is obtained, as shown in Figure S1e.


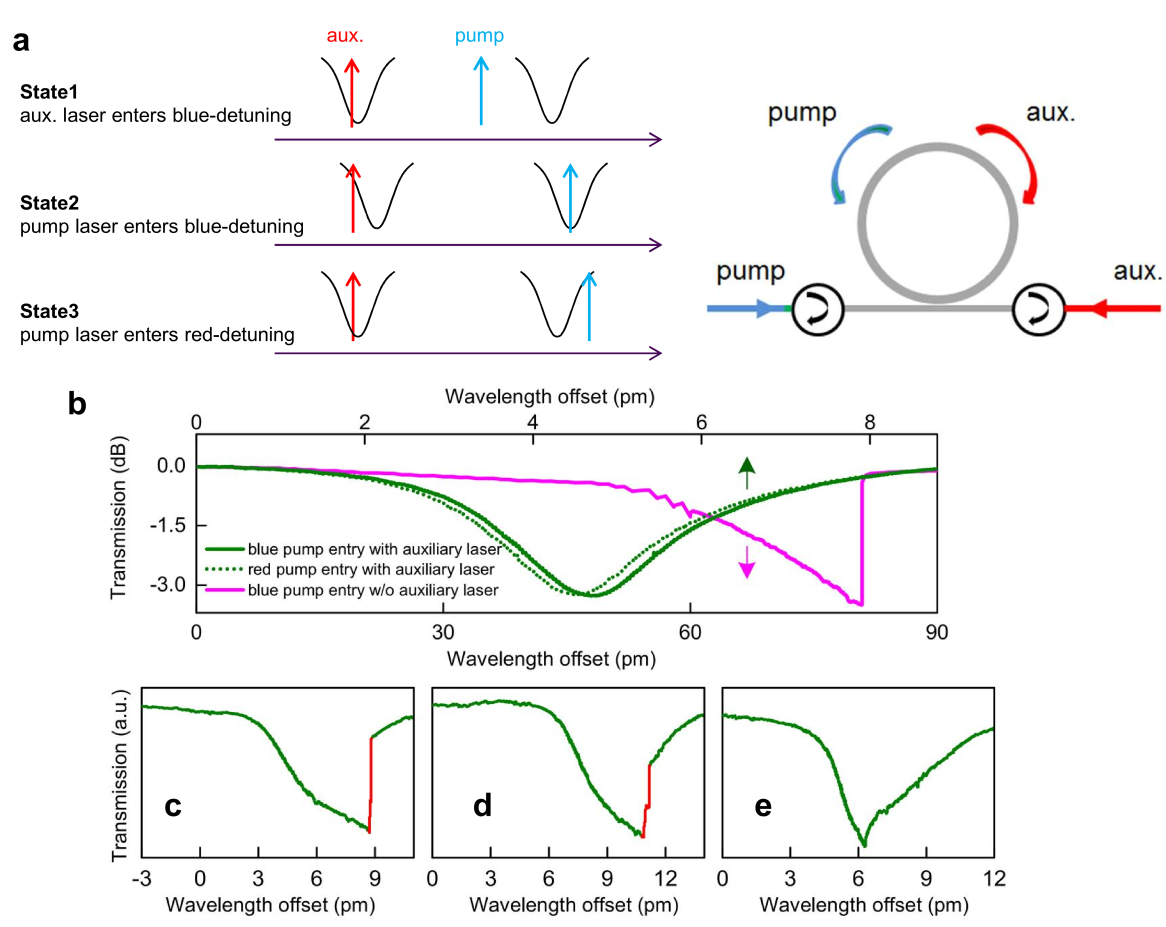


**Figure S1 | Benchmarking of auxiliary laser assisted comb generation method. a,** Schematics of the auxiliary laser heating method; **b,** Comparison of pump transmission with and without auxiliary laser. Pump power is set at 27.0 dBm, under parametric oscillation threshold. **c-e**, Pump transmission for: **c,** 29.0 dBm; **d,** 30.0 dBm; and **e,** 31.0 dBm launched auxiliary power, the pump power is fixed at 30.0 dBm. Smooth pump transmission shown in panel **e** with minor thermal hysteresis implies appropriate auxiliary laser configuration. Discrepancy from Lorentz shape (comparing with panel **b**) is due to Kerr nonlinear dynamics.

**II.** **Beat note, phase noise and Allan deviation measurements of the DKS combs**

The approximately 191.31 GHz mode-spacing of the frequency comb obtained in our study hinders direct beat note characterization. Therefore we utilize cross-phase modulation (XPM) to measure the comb spacing via nonlinear modulation sidebands. The soliton microcomb and a pulsed laser (repetition-rate of ≈ 11.45 GHz with 10 ps pulse widths) are combined and launched into a 500 m highly nonlinear fiber (HNLF), where the picosecond pulse train imposes considerable XPM on each comb line and generates multiple nonlinear modulation sidebands covering approximately the original comb spacing (≈ 191.31 GHz), as shown in Figure S2a. Then detectable beat notes can be produced by the overlapped XPM sidebands from two adjacent comb lines [S2]. As shown in Figure S2b, the recorded beat note is much narrower than the free-running pump laser linewidth, confirming the excellent coherence of our DKS microcomb. For comparison, the beat note of a high-noise Kerr comb is also measured and presented in Figure S2c, exhibiting much higher noise as wide as the cavity resonance.

Furthermore, since our dual-driven scheme can largely suppress the thermal shift of the cavity resonances during pump detuning scan, now we can accurately measure the relationship between comb spacing and pump detuning [S3, S4], aided by the above XPM scheme. Such measurement shows a ratio of ≈ 15 kHz MHz^-1^ between comb spacing and pump detuning, which is much bigger than prior measured phase-locked Kerr comb without DKS formation [S5].


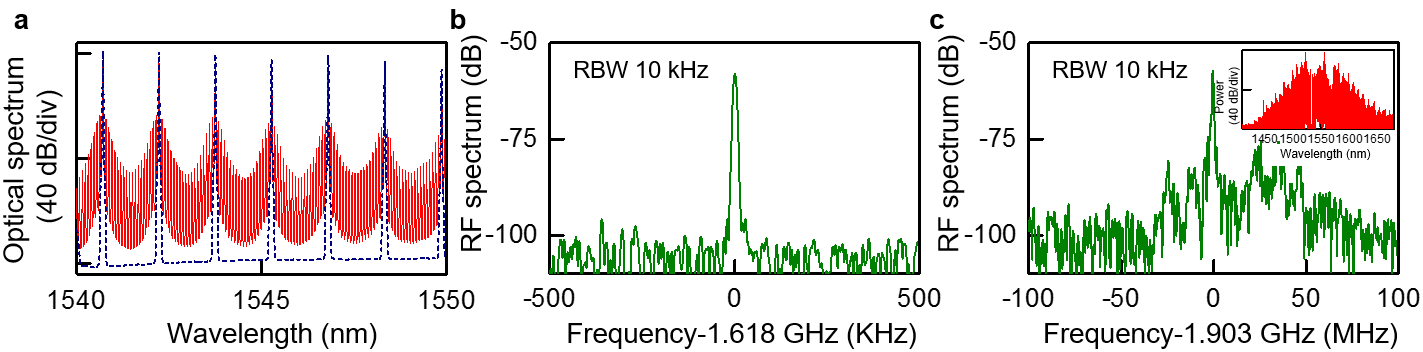


**Figure S2 |** **Comb** **beat note measurement via cross-phase modulation spectral broadening. a,** DKS comb spectrum before (blue dashed-line) and after (red solid-line) XPM. **b,** DKS comb beat note generated by two interleaved XPM sidebands spaced by ≈1.618 GHz. **c,** Beat note of a high-noise Kerr comb, generated by two interleaved XPM sidebands spaced by ≈ 1.903 GHz. Inset shows the corresponding high-noise comb spectrum.

We also measured the beat note between comb lines (corresponding to state i, ii, iii in Fig. 1 of the main text) and a narrow linewidth CW laser (NKT Basik E15 with 1 kHz linewidth), as show in Figure S3 below. All beat notes exhibit narrow linewidth, limited only by the coherence between the CW laser and the pump laser. Neither additional linewidth broadening of the comb lines relative to the pump laser nor multiple beat notes are observed, confirming that the comb lines exhibit a similar level of phase noise as the pump. In comparison, the high noise comb state shows a much wider beat note with the CW laser, implying much higher phase noise. Single-sideband phase noise measurements of the beat notes between comb lines and a narrow linewidth laser are also conducted as shown in Figure S3b. The comb lines exhibit similar phase noise as the pump laser, indicating that no additional noises are added to comb State i, ii, and iii. Moreover, dashed lines show the direct beat note phase noise between adjacent comb lines, by reducing the ~190 GHz comb spacing to 19.9 MHz using higher order phase modulation at frequency 38.253 GHz (see Figure S4 below). Much lower phase noises obtained from comb-lines-beating than comb-laser-beating indicate that the comb lines in State ii and State iii are of high coherences, confirming multiple and singlet soliton generations, respectively.


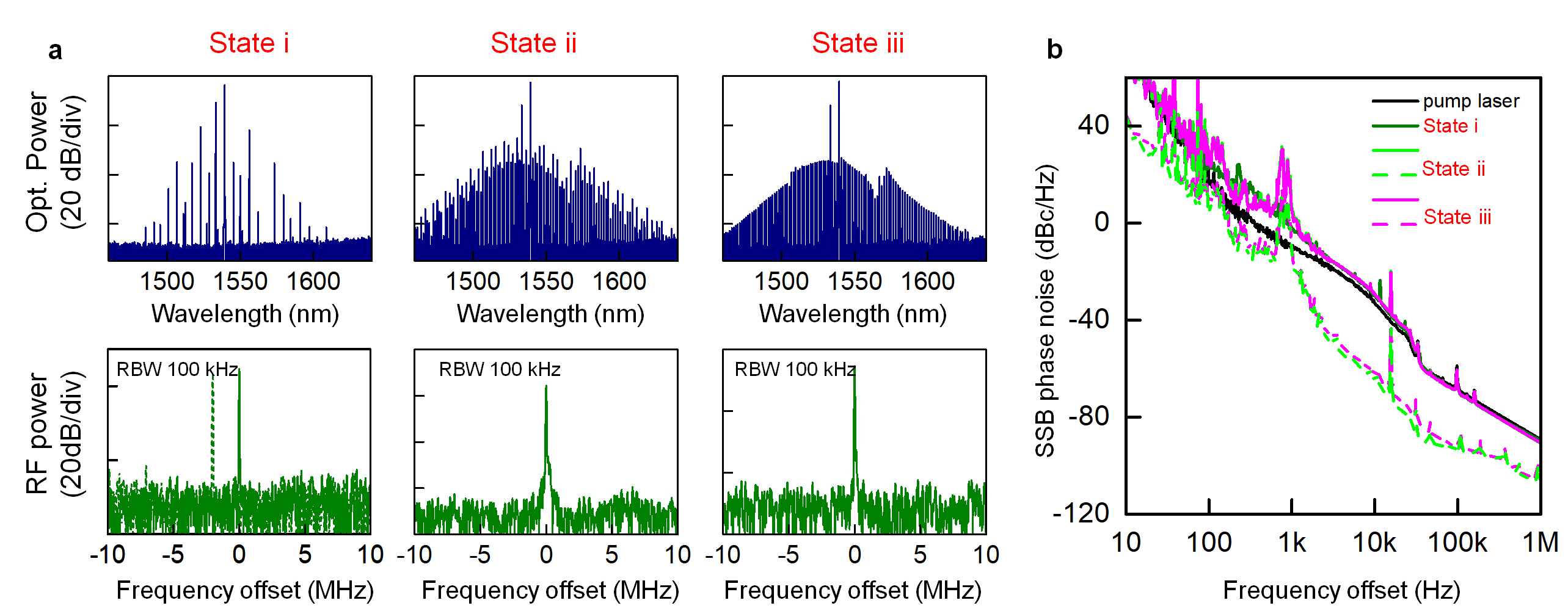


**Figure S3 | a,** DKS comb spectra (upper) and beat note spectra between comb lines and a narrow linewidth laser (lower), for different comb states. State i, ii, and iii all show narrow linewidth, implying that these combs have similar level of phase noise as the pump. The dashed green line in lower left panel shows the beat note between the pump and narrow linewidth CW laser. **b,** single-sideband phase noise measurements of the beat notes between comb lines and a narrow linewidth laser (solid lines), black line shows the phase noise of the pump laser itself beating with the narrow linewidth laser. Since our pump laser is not stabilized (i.e., 500 kHz linewidth), the measured phase noises are relatively high. Nevertheless, we can see that the comb lines exhibit similar phase noises as the pump laser, indicating that no additional noises are added to comb State i, ii, and iii. Minor additional noise (small peaks near 1kHz) is due to fluctuations of free-space pump coupling. Moreover, dashed lines show the beat note phase noises between adjacent comb lines, by reducing the ~190 GHz comb spacing to 19.9 MHz using higher order phase modulation at frequency 38.253 GHz (see Figure R3 below). Much lower phase noises obtained from comb-lines-beating than comb-laser-beating indicate that the comb lines in State ii and State iii are of high coherences, confirming multiple and singlet soliton generations in State ii and State iii, respectively.

Moreover, we have added Allan deviation measurement of the comb repetition rate, by reducing the ~190 GHz comb spacing to 19.9 MHz using higher order phase modulation at frequency 38.253 GHz, as shown in Figure S4. It is seen that our comb exhibits reasonably nice frequency stability in the free-running condition. The residual frequency variation is mainly due to pump laser frequency drifts. Fully stabilizing the pump laser to a more stable reference source (e.g., a stabilized fiber frequency comb) can greatly improve the Allan deviation of comb spacing [S5], but is beyond the scope of this article.


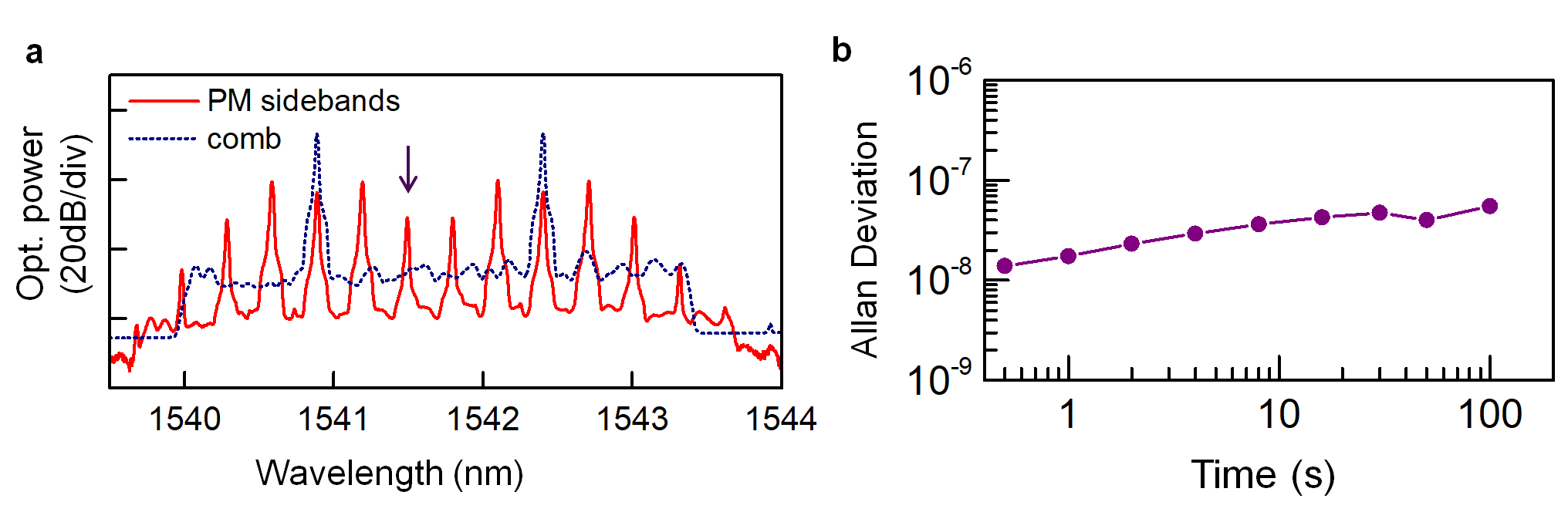


**Figure S4 |** **Allan deviation measurement of the single DKS comb. a,** Reducing the ~190 GHz comb spacing to 19.9 MHz by using higher order phase modulation at frequency 38.253 GHz. The arrowed two overlapping sidebands are used for Allan Deviation measurement. **b,** Allan deviation as the function of gated time of free-running single DKS comb.

**III. Residual perturbation caused by backscattered auxiliary laser**

It is seen in Figure 1d of the main text that the generated DKS spectrum contains a backscattered component of the auxiliary laser, which is much smaller (≈ 15 dB smaller) than the pump laser. As described in the main text, the auxiliary laser and the pump laser have large detuning offset (especially in DKS states), thus the nonlinear perturbation from the backscattered auxiliary laser has only minor perturbation to the DKS comb dynamics. In particular, as shown in Figure S5b, the residual perturbation of the backscattered auxiliary laser is a small narrow-band noise sideband in the comb beat note spectrum. This noise is incurred by the incoherent heterodyne beating (≈ 550 MHz) between the backscattered auxiliary laser and the generated comb line in the same resonance, which then imposes a global nonlinear phase modulation to all comb lines, causing a narrow-band noise to the DKS microcomb [S6]. To validate our analysis, we used an orthogonally polarized auxiliary laser (i.e., fundamental TE_00_ mode near 1544.6 nm) with respect to the pump and realized cavity thermal compensation in another polarization mode [S7]. Since the offsets between different polarization modes are usually larger, the beating oscillation between the auxiliary laser and the adjacent comb line is significantly higher than the bandwidth of photodetector and spectrum analyzer (≈ 26 GHz). Thus the noise component disappears from the RF spectrum, as shown in Figure S5b lower panel.

Moreover, we conduct similar measurement for a L-band (1576.8 nm) pumped DKS comb with 19.7 GHz repetition rate, and set a co-polarized auxiliary laser still at C-band (≈ 1539.3 nm). In such case the auxiliary laser only overlaps with the low power comb section, and the beating between the auxiliary laser and corresponding comb line becomes negligible, consequently no noise component is observed, as shown in Figure S5 bottom panel.


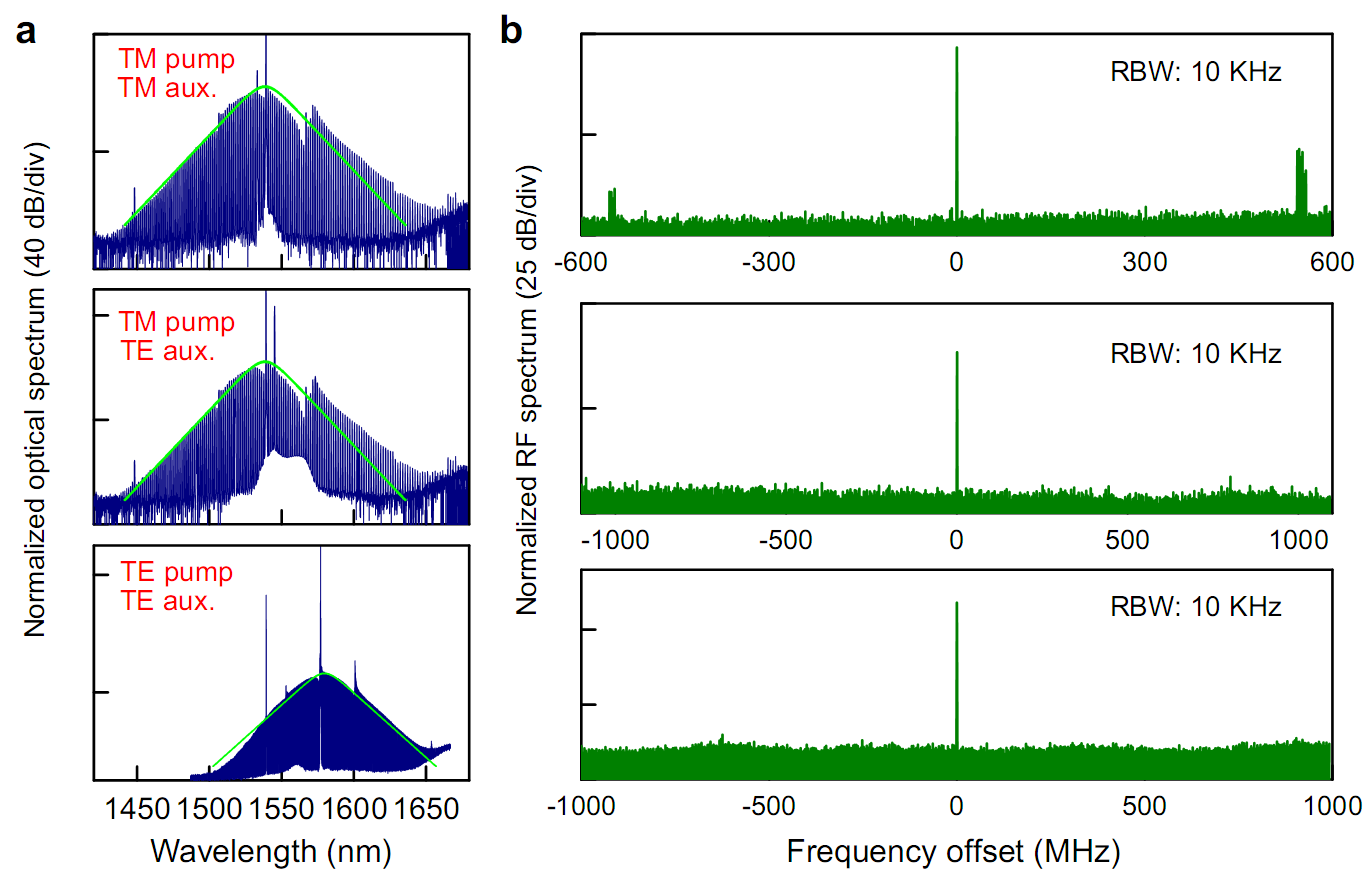


**Figure S5 |** **Residual perturbation caused by the backscattered auxiliary laser to DKS microcomb.** **a,** DKS comb spectrum and **b,** Beat note spectrum for co-polarized pump and auxiliary laser (upper panels)；orthogonally-polarized pump and auxiliary laser (middle panels); co-polarized pump and auxiliary laser, but with reduced overlapping between auxiliary laser and comb spectrum (bottom panels).

**IV. Autocorrelation measurement of DKS microcombs**

Figure S6a shows the setup for autocorrelation measurement. After the main setup, the pump and auxiliary laser are removed using two cascaded fiber Bragg gratings (FBGs). A liquid-crystal-on-silicon (LCOS) based wavelength-selective switch (WSS, Finisar WaveShaper 4000s) is utilized to choose out the C-band comb spectrum and compensate the dispersion induced within fiber patch cord. The filtered DKS comb component is then sent into a second-harmonic generation (SHG)-based autocorrelator (Femtochrome FR-103XL) after being amplified to 15.0 dBm using a C-band EDFA. By adjusting the WSS dispersion value to -1.10 ps nm^-1^, high-contrast autocorrelation traces are obtained, as shown in Figure S6b. Due to the distortion induced by spectrum truncation and EDFA noise, a pedestal about 20% of the total autocorrelation amplitude is introduced, and the measured pulse width is artificially broadened to about 650 fs (assuming *Sech*-shaped soliton pulse). However, such pulse distortion does not compromise the validation of DKS generation. Also, Figure 6b exemplifies the autocorrelation traces when WSS dispersion value is not properly configured. It is seen that autocorrelation traces degrade into flat waveforms for zero and -5.0 ps nm^-1^ dispersion added by the WSS, evidencing that the intracavity waveforms are ultrashort pulses instead of periodic- non-pulse waveforms (which are insensitive to dispersion).

Moreover, we also measured the autocorrelations of multiple-DKS combs. Figure S6c and S4d present the autocorrelation traces for DKS combs associated with two DSK pulses but with different pulse allocations. In particular, we see two peaks in the autocorrelation trace when two DKS pulses are evenly separated (Figure S6c); while three peaks appear in the autocorrelation trace when two DKS pulses are unevenly separated (Figure S6d). These results further confirm short DKS pulses formation in the cavity.

**
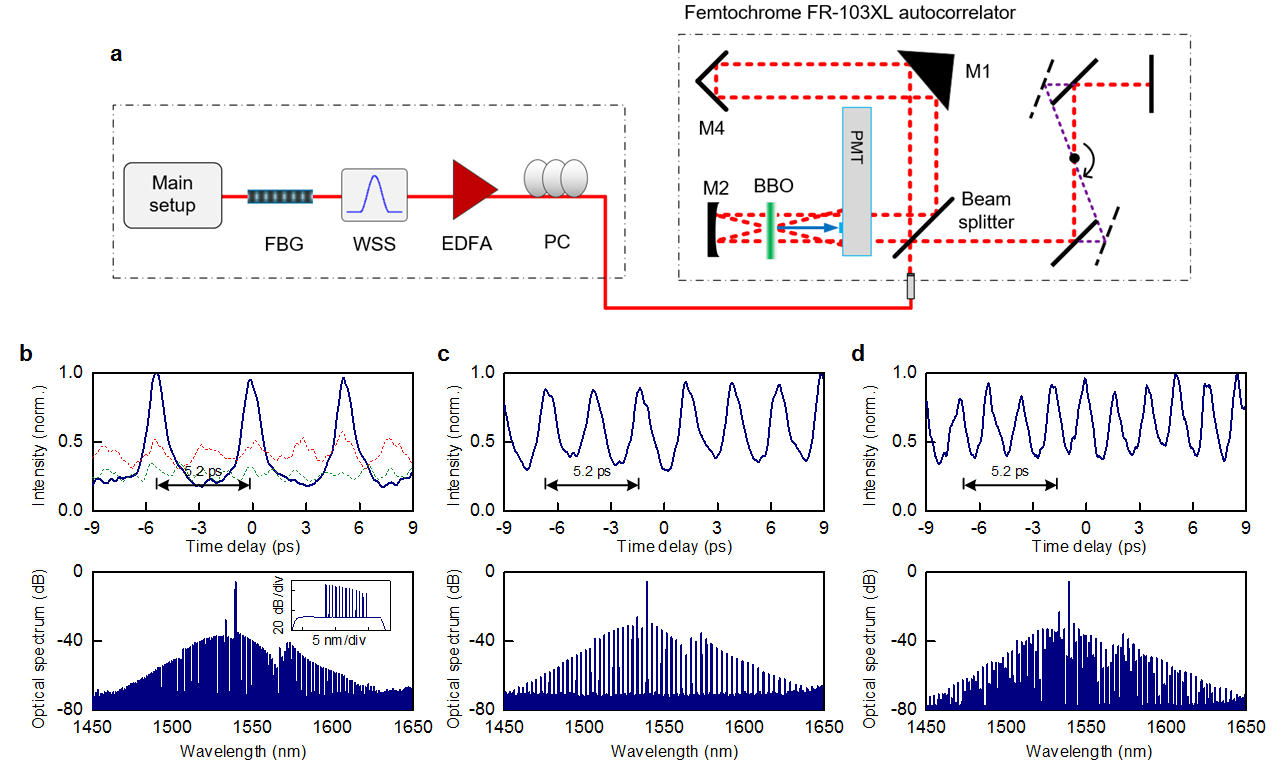
**

**Figure S6 |** **Autocorrelation measurement of DKS microcombs.** **a,** Experimental setup. BBO: Barium Boron Oxide; PMT: photomultiplier tube. **b,** Measured autocorrelation (upper) and spectrum (lower) for singlet DKS microcomb (blue-solid line). The WSS is set to have a dispersion of -1.1 ps nm^-1^ to generate finest pulse. AC trances are also measured when the WSS dispersion value is set to 0 ps nm^-1^ (red-dotted line) and -5.0 ps nm^-1^ (green-dotted line). The lower panel inset shows the filtered and amplified spectrum used for autocorrelation measurement. **c,** Measured autocorrelation (upper) and spectrum (lower) for microcombs with two evenly separated DKS pulses. **d,** Measured autocorrelation (upper) and spectrum (lower) for microcomb with two unevenly separated DKS pulses.

**V. Analytical solutions of the diminishing soliton regime**

The LLE model shown as Eq. (1) in the main text can be transformed to the dimensionless form [S8]:

$i\frac{\partial\Psi}{\partial\tilde{t}}+\frac{1}{2}\frac{\partial^{2}\Psi}{\partial\tilde{\tau}^{2}}+\left|\Psi\right|^{2}\Psi=\text{(}\zeta_{0}-i\text{)}\Psi+ih$ (S1)

$\Psi=\sqrt{\frac{\gamma L}{\alpha}}E$, $h=\sqrt{\frac{\theta\gamma LP_{\text{in}}}{\alpha^{3}}}$, $\tilde{t}=\frac{\alpha t}{T_{R}}$, $\tilde{\tau}=\tau\sqrt{-\frac{\alpha n_{0}}{cT_{R}\beta_{2}}}$,$\zeta_{0}=\frac{\delta_{0}}{\alpha}<\frac{\pi^{2}h^{2}}{8}$, and $\delta_{0}\text{=}T_{R}\text{(}\omega_{0}-\omega_{\text{p}}\text{)}$, *n*_0_ is the refractive index, *c* is the speed of light, *ω*_0_ is the resonant frequency of the pumped mode, *ω*_p_ is the pump angular frequency, and *P*_in_ is the pump power. Using Lagrangian perturbation method, the soliton solution can be approximated for the smaller detuning *ζ*_0_ [S8]:

$\Psi=C+S(\tilde{\tau})$ (S2)

$B_{1}\text{=}B_{0}-\frac{5\pi}{8\zeta_{0}}\mathrm{h}\text{cos}\left(\chi\right)$ (S3)

$B_{2}\text{=}B_{0}-\frac{\pi}{4\zeta_{0}}\mathrm{h}\text{cos}\left(\chi\right)$ (S4)

(S5)

$C=-i\frac{h}{\zeta_{0}}+\frac{h}{\zeta_{0}^{2}}+i\frac{h}{\zeta_{0}^{3}}-\frac{h+ih^{4}}{\zeta_{0}^{4}}+O\text{(}\frac{1}{\zeta_{0}^{5}}\text{)}$,$B_{0}\text{=}\sqrt{2\zeta_{0}}$, and. Using Eq. (S2-S5), we can analytically calculate the soliton energy and waveform evolution versus pump detuning, using the experimental parameters (listed in Table S1). As shown in Figure S7, remarkable agreement between analytical and numerical result (Figure 2b-c in the main text) is achieved, further supporting the existence of the diminishing soliton regime.

**Table S1** | Parameters used in analytical calculations.

| parameters | Value |
| --- | --- |
| *γ* | 1.0 w^-1^∙m^-1^ |
| *β*_2_ | -20×10^-27^ s^2^ m^-1^ |
| *n*_0_ | 2.4 |
| *L* | 741.4 µm |
| *α* | 0.006 |
| *θ* | 0.006 |
| *D*_1_ | 191 GHz |


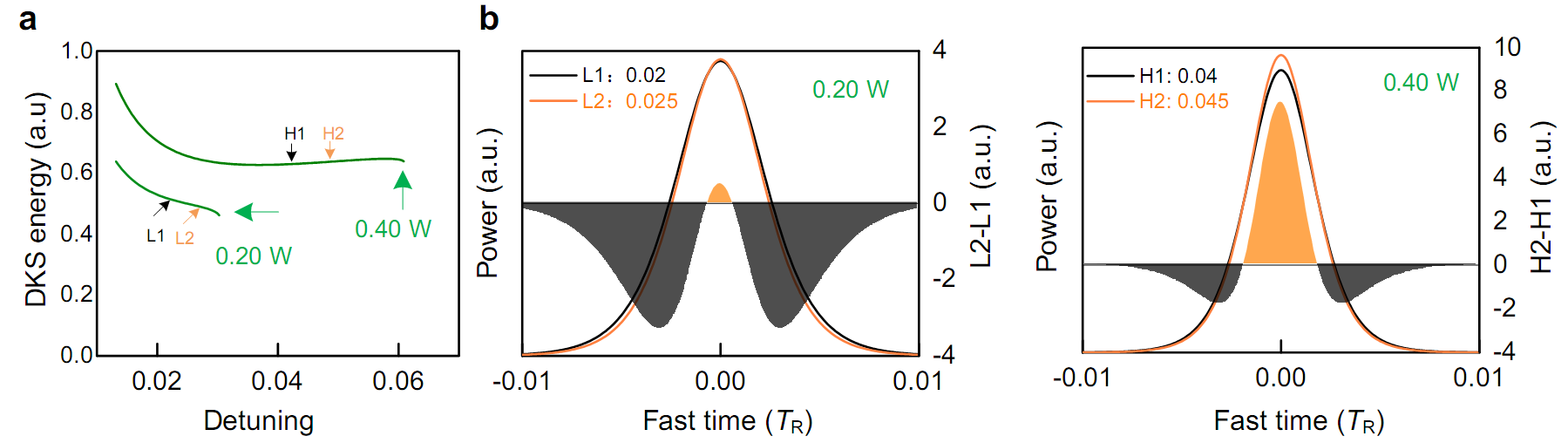


**Figure S7 |** **Analytical calculation of DKS existence regime.** **a,** Theoretically calculated DKS existence range in the increasing and diminishing soliton regime respectively, illustrating remarkable agreement with the numerical results shown in Figure 2b of the main text. **b**, Close-up soliton waveforms versus pump detuning in the diminishing (left) and increasing (right) soliton regime.

**VI. Experimental evidence of soliton energy evolution change**

As shown in Figure 2b of the main text, the slope of DKS energy (average power) changes for different pump powers. Figure S8 shows direct experimental evidences of this phenomenon. It is seen that with increasing pump powers from 21.0 dBm to 23.5 dBm (estimated on-chip pump power), the slope of comb power evolution increases from negative value towards positive value, supporting our theoretical analysis in Figure 2b. Due to the moderate *Q*-factor of our microcavity and the power limitation of our pump EDFA, we do not reach into the increasing soliton regime with absolute positive slope comb power evolution (seen in prior published DKS studies [S7, S8]).


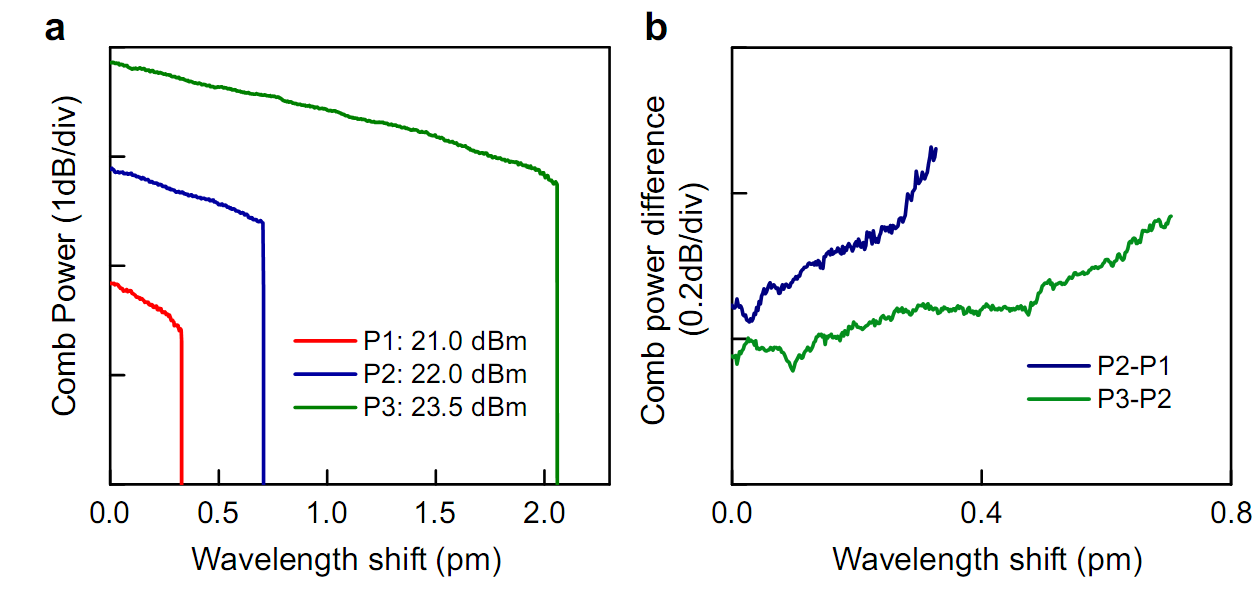


**Figure S8 |** **Experimental evidences of soliton energy evolution change.** **a,** Singlet DKS microcomb power evolutions generated by different pump powers. **b,** Differences between the traces in panel **a**, for better visualization of comb power evolution slope change.

**VII. Influence of thermal effect and auxiliary laser on DKS dynamics**

Here we present detailed LLE numerical simulations on the influence of thermal effect and auxiliary laser on DKS dynamics. Figure S9 shows the DKS formation dynamics via blue-side pump entry. With both thermal effect and auxiliary laser applied, the cavity resonance can be thermally pushed to about 0.16 (～4.86 GHz) by the auxiliary laser in advance. Afterwards, when the pump laser enters the preheated cavity, DKS microcomb can be generated, qualitatively identical with the dynamics obtained by conventional LLE model without thermal effect (black traces), indicating that auxiliary laser can thoroughly separate thermal effect from Kerr nonlinear dynamics inside the microcavity. In comparison, when thermal effect is included but auxiliary laser is not (green traces), the pump power rapidly vanishes once it enters into the red-detuning regime, and no DKS is observed.


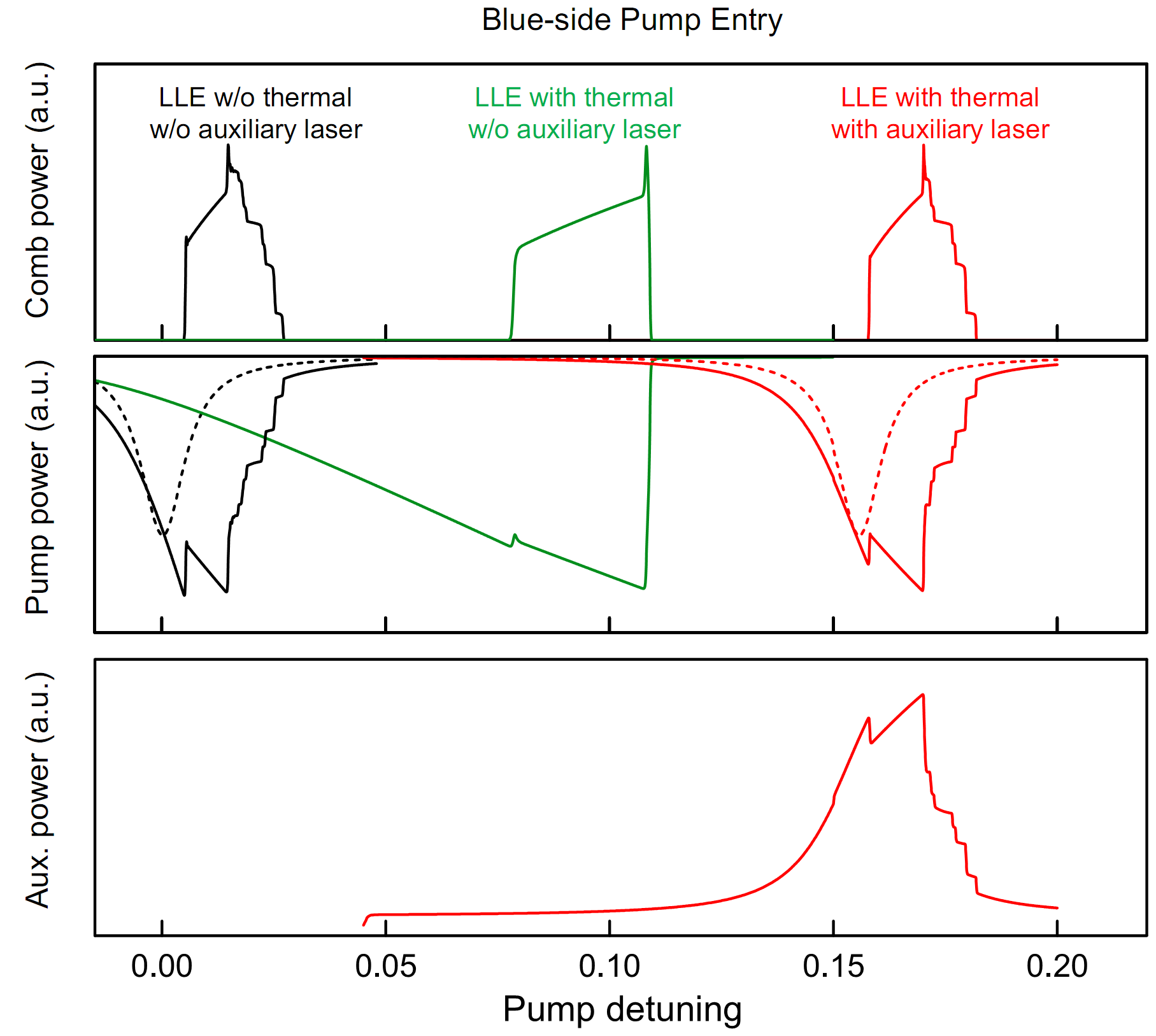


**Figure S9 |** **LLE simulated DKS formation with and without cavity thermal effect and auxiliary laser.** In the simulation, pump power is 0.25 W and auxiliary laser is 0.5 W. The auxiliary laser is first tuned into resonance and stopped at *δ*_aux_ = 0.12, which thermally pushes the cavity resonance by a detuning value of 0.16 (～4.86 GHz, including Kerr effect induced resonance shift), as illustrated by the red-dashed line. Afterward, the pump laser is tuned into resonance and it stably generates DKS dynamics. Black lines present the simulation results when thermal effect and auxiliary laser is not included, which are quite identical the red traces, indicating that the thermal effect incurred by the pump and auxiliary laser cancels out each other. In comparison, when the thermal effect is included but the auxiliary laser is absent, the pump laser cannot stabilize on the red-detuning regime and no DKS is generated in this case (green lines).


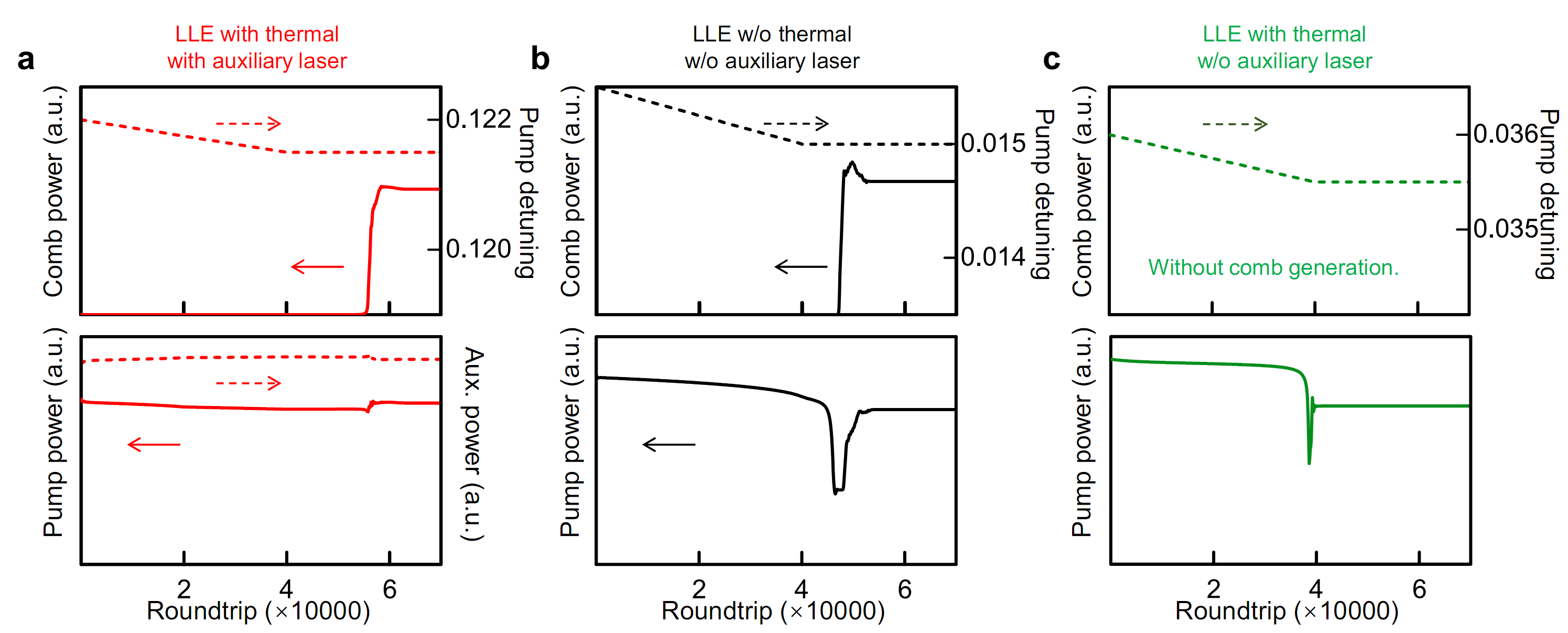


**Figure S10 |** **LLE simulated DKS burst with and without cavity thermal effect and auxiliary laser. a,** Simulated DKS burst dynamics with thermal effect and auxiliary laser. Power configurations are identical with Figure S7. The auxiliary laser is first tuned into resonance and stopped at *δ* _aux_ = 0.10. Afterward, the pump laser is tuned into the preheated cavity from the red detuning regime, as shown in the upper panel. **b,** DKS burst dynamics without thermal effect, which is similar with the result in panel **a**, indicating that the thermal effects incurred by the pump and auxiliary laser cancel out each other. The deep dip of pump power is caused by Kerr nonlinear resonance shift, which is not seen in panel **a** since the auxiliary laser also offsets Kerr-induced resonance shift [S9]. **c,** When thermal effect is included but the auxiliary laser is absent, the pump laser cannot stabilize on the red detuning and no comb is generated in this case.

Figure S10 shows the simulated DKS burst dynamics with and without cavity thermal effect. Similarly, when thermal effect is included but simultaneously offset by the auxiliary laser, the numerically resolved burst dynamics is qualitatively identical with the dynamics obtained by pure LLE model. In contrast, when the thermal effect is included but the auxiliary laser is absent, the pump laser does not stabilize on the red-detuning regime and no DKS is generated in this case.

**VIII. Auxiliary laser assisted extension of DKS existence range**

As shown in Equations S1 and S2 of the main text, the counter-propagating comb and auxiliary laser impose a slowly-varying nonlinear detuning shift to each other via Kerr-nonlinearity XPM, subjecting to their average power *P*_comb_ and *P*_aux_ [S9]. Specifically, when pump laser enters the red-detuning regime for DKS formation, XPM induced by the auxiliary laser (2*iγLP*_aux_) counter balances the increase of pump detuning *δ*_pump_ and the decrease of self-phase modulation of the pump laser itself (contained in 2*iγL|E*_comb_*|*^2^), which in turn retards the increase of effective pump detuning. That is to say, XPM from the auxiliary laser can extend the DKS existence range in a similar fashion as in thermal dynamics. The above dynamics is illustrated via LLE modeling, as shown in Figure S11. It is seen that with the XPM terms in Eq. S1 and S2, the effective detuning has a relatively flatter section as the function of pump detuning (i.e. pump laser frequency) than without XPM, giving rise to an extended DKS existence range.


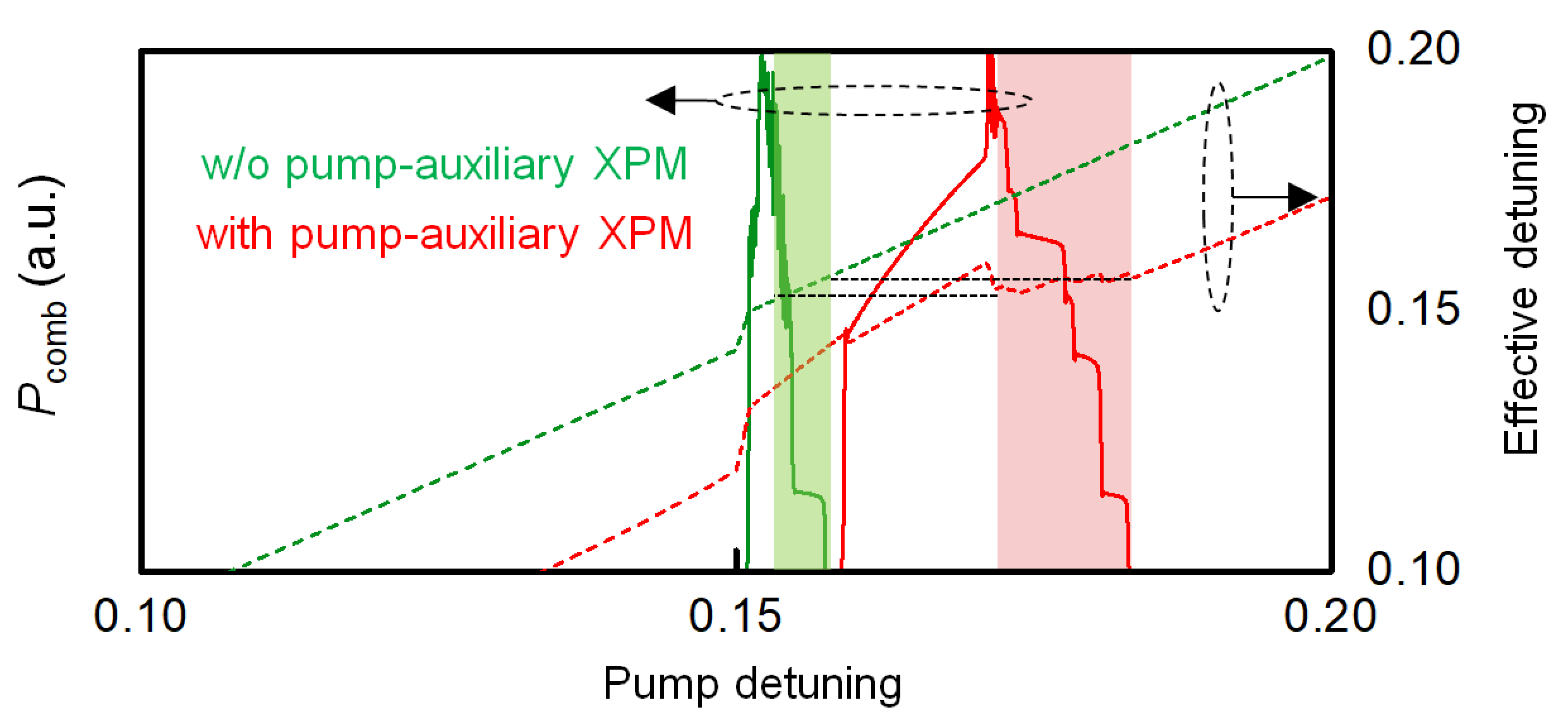


**Figure S11 |** **Extension of DKS existence range aided by pump-auxiliary XPM.** LLE modeled comb power evolution with (red solid line) and without (green solid line) the nonlinear terms associated with mutual XPM between the pump and auxiliary laser. Dashed green line to the right-*y* axis denotes the pump detuning *δ*_pump_, dashed red line shows the effective pump detuning calculated as *δ*_pump_−2*iγLP*_aux_.

**Supplementary References**

[S1] Grudinin, I., Lee, H., Chen, T., & Vahala, K. J. Compensation of thermal nonlinearity effect in optical resonators. Opt. Express 19, 7365-7372 (2011).

[S2] Del’Haye, P., Papp, S. B., & Diddams, S. A. Hybrid electro-optically modulated microcombs. Phys. Rev. Lett. 109, 263901 (2012).

[S3] Li, J., Diddams, S. A., & Vahala, K. J. Pump frequency noise coupling into a microcavity by thermo-optic locking. Opt. Express 22, 14559-14567 (2014).

[S4] Lucas, E., Guo, H., Jost, J. D., Karpov, M., & Kippenberg, T. J. (2017). Detuning-dependent properties and dispersion-induced instabilities of temporal dissipative Kerr solitons in optical microresonators. Physical Review A, 95(4), 043822.

[S5] Huang, S. W., Yang, J., Lim, J., Zhou, H., Yu, M., Kwong, D. L., & Wong, C. W. (2015). A low-phase-noise 18 GHz Kerr frequency microcomb phase-locked over 65 THz. Scientific reports, 5, 13355.

[S6] Herr, T., Hartinger, K., Riemensberger, J., Wang, C. Y., Gavartin, E., Holzwarth, R., Gorodetsky, M.L., & Kippenberg, T. J. (2012). Universal formation dynamics and noise of Kerr-frequency combs in microresonators. Nature Photonics, 6(7), 480.

[S7] Li, Q., Briles, T. C., Westly, D. A., Drake, T. E., Stone, J. R., Ilic, B. R., Diddams, S.A., Papp, S.B., & Srinivasan, K. (2017). Stably accessing octave-spanning microresonator frequency combs in the soliton regime. Optica, 4(2), 193-203.

[S8] Guo, H., Karpov, M., Lucas, E., Kordts, A., Pfeiffer, M. H., Brasch, V., Lihachev, G., Lobanov, V.E., Gorodestsky, M.L., & Kippenberg, T. J. (2017). Universal dynamics and deterministic switching of dissipative Kerr solitons in optical microresonators. Nature Physics, 13(1), 94.

[S9] Del Bino, L., Silver, J. M., Stebbings, S. L., & Del'Haye, P. (2017). Symmetry breaking of counter-propagating light in a nonlinear resonator. Scientific Reports, 7, 43142.
